# Adaptive intertidal seed-based seagrass restoration in the Dutch Wadden Sea

**DOI:** 10.1371/journal.pone.0262845

**Published:** 2022-02-09

**Authors:** Laura L. Govers, Jannes H. T. Heusinkveld, Max L. E. Gräfnings, Quirin Smeele, Tjisse van der Heide

**Affiliations:** 1 Conservation Ecology Group, Groningen Institute for Evolutionary Life Sciences (GELIFES), University of Groningen, Groningen, The Netherlands; 2 Department of Aquatic Ecology and Environmental Biology, Institute for Water and Wetland Research (IWWR), Radboud University, Nijmegen, The Netherlands; 3 Department of Coastal Systems, Royal NIOZ and Utrecht University, Den Burg, The Netherlands; 4 The Fieldwork Company, Groningen, The Netherlands; 5 Natuurmonumenten, Paterswolde, The Netherlands; University of Sydney, AUSTRALIA

## Abstract

Seagrasses form the foundation of many coastal ecosystems but are rapidly declining on a global scale. The Dutch Wadden Sea once supported extensive subtidal seagrass meadows that have all disappeared. Here, we report on the setbacks and successes of intertidal seed-based restoration experiments in the Dutch Wadden Sea between 2014–2017. Our main goals were to 1) optimize plant densities, and 2) reduce seed losses. To achieve our goals, we conducted research-based, adaptive seagrass (*Zostera marina*) restoration, adjusting methods yearly based on previous results. We applied various seeding methods in three subsequent years–from Buoy Deployed Seeding (BuDS), and ‘BuDS-in-frame’ in fall, to a newly developed ‘Dispenser Injection Seeding’ (DIS) method. Our adaptive experimental approach revealed high seed losses between seeding and seedling establishment of the BuDS methods (>99.9%), which we mitigated by controlled harvest and storage of seeds throughout fall and winter, followed by DIS-seeding in spring. These iterative innovations resulted in 83 times higher plant densities in the field (0.012 to 1.00 plants m^-2^) and a small reduction in seed loss (99.94 to 99.75%) between 2015–2017. Although these developments have not yet resulted in self-sustaining seagrass populations, we are one step closer towards upscaling seagrass restoration in the Dutch Wadden Sea. Our outcomes suggest that an iterative, research-based restoration approach that focuses on technological advancement of precision-seeding may result in advancing knowledge and improved seed-based seagrass restoration successes.

## Introduction

Seagrasses form the foundation of many coastal ecosystems and support millions of people living in coastal areas across the planet [1, 2]. These vital ecosystems are, however, globally disappearing as a result of anthropogenic activities [3, 4] such as eutrophication, habitat-destruction and climate-induced heat waves [5–7]. To halt and reverse these losses, many attempts to restore seagrass meadows are currently undertaken [8, 9]. About 50% of these attempts concern *Zostera marina* (eelgrass), the most common habitat-forming seagrass species in the Northern Hemisphere [10]. However, seagrass restoration is challenging and success rates are generally low. Hence, rapid advancements in this field are currently needed [11].

The Dutch Wadden Sea once harboured extensive seagrass meadows. Before the 1930s, the Dutch Wadden Sea was covered by around 15,000 hectares of subtidal, perennial *Z*. *marina* beds, which disappeared due to the wasting disease which coincided with the construction of a major dam (‘de Afsluitdijk’) that reduced light availability to submerged plants [12, 13]. Although intertidal beds survived, they have mostly withered away, most likely due accumulation of stressors such as eutrophication [14], increased bioturbation [15, 16] and increased sediment dynamics resulting from large-scale modification of the landscape [17, 18]. Currently, the Dutch Wadden Sea contains only a meagre 11.3 ha of seagrass of which only 0.5 ha with a plant cover higher than 20% [19]. Most of this seagrass is perennial *Zostera noltii* with a very rare occurrence of *Z*. *marina*. This low number contrasts with the total seagrass area in the northern Wadden Sea (Germany, Denmark), where intertidal seagrass meadows rapidly recovered in recent decades. Here, over 200 km^2^ of intertidal seagrass is thriving in mixed meadows of perennial *Z*. *noltii* and annual *Z*. *marina* [19–22].

Due to its high ecological importance, several intertidal restoration attempts have been conducted in the Dutch Wadden Sea, starting in the 1950s [12], followed by more research-based attempts from 1990–2005, that explored both the use of plants and seeds as donor material [23–25]. The next episode in Dutch Seagrass Restoration (2010–2013) involved a “Let’s try” [26] attitude, initiated by Rijkswaterstaat (Dutch implementation agency of the Ministry of Infrastructure and Water Management) and an NGO (de Waddenvereniging). Large-scale seed-based restoration attempts (1 ha site^-1^, 3 sites) were conducted in two consecutive years (2011 and 2012) using buoy deployed seeding (BuDS) [27, 28], targeting intertidal, annual *Z*. *marina*. Plants emerged after seeding by BuDS across large surface areas (~300 ha), indicating large seeded areas, but plant densities were very low (<1% cover) [29].

**Fig 1:**
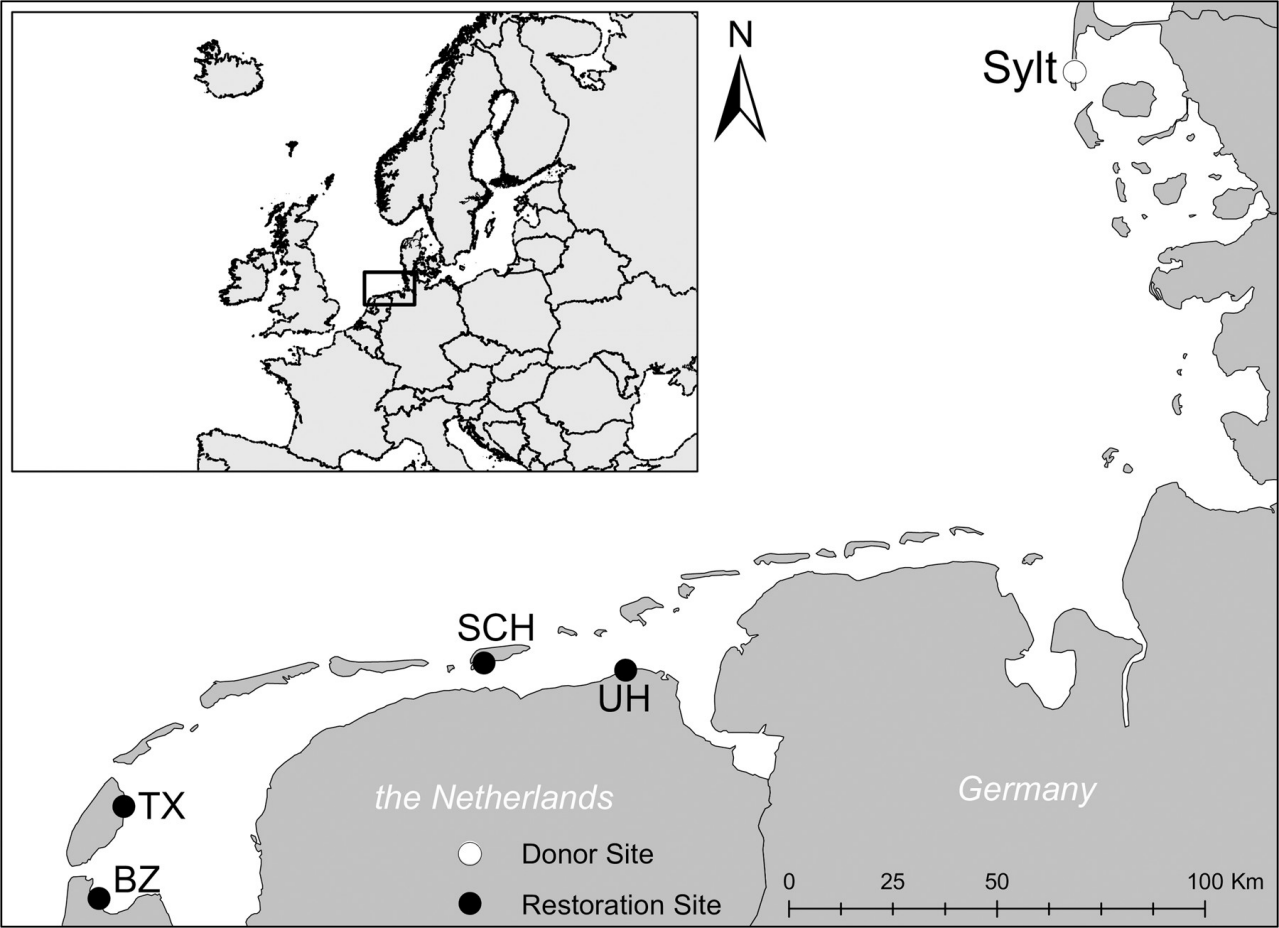
Location maps. (A) Location of restoration sites and donor site in North Western Europe, (B) Locations of restoration sites (black dots) and donor site (Sylt, white dot) in the Dutch Wadden Sea. Abbreviations of donor sites stand for: BZ = Balgzand, TX = Texel, Vlakte van Kerke, SCH = Schiermonnikoog, UH = Uithuizen. Maps were obtained through Eurostat/GISCO, and are freely available for non-commercial use.

Here, we describe the results of seed-based intertidal seagrass restoration trials in the Dutch Wadden Sea between 2014–2017, which built upon earlier work from 2011–2012 and 1990–2006 [23]. Rather than targeting large-scale restoration, this episode (2014–2017, or ‘project’) focused on developing the tools needed for large-scale restoration by identifying and tackling vital restoration bottlenecks through an iterative, experimental approach. We conducted research-based, adaptive seagrass restoration focusing on technological advancement to improve restoration successes. This meant that results were evaluated yearly and methods adapted accordingly. This approach yielded new methods and techniques through which we aimed to 1) increase restoration success in terms of plant densities (m^-2^) and 2) reduce seed losses. Target plant densities were >10 plants m^-2^ to enable self-facilitation [25], with seed losses <99%, as plants typically produce around 100 seeds plant^-1^, yielding negative population growth when loss rates exceed 99% [29]. Restoration experiments were conducted on five sites in the Dutch Wadden Sea (Fig 1), using seeds from a German seed-donor site.

## Materials and methods

### Site description

The Wadden Sea encompasses an area of around 15,000 km^2^, spanning from the Netherlands to Denmark (Fig 1). It is a UNESCO world heritage area and the world’s largest unbroken system of intertidal mud and sand flats [30]. Despite its recognized status, the Wadden Sea is heavily modified by human activities, both by resource depletion [16, 31] and large-scale modification of the landscape by the construction of dams and dikes [32]. This study focuses on restoration experiments in the intertidal Dutch Wadden Sea (2500km^2^).

### Restoration sites

Restoration sites varied among years (2014–2017). Initially (2014), four sites were selected (Fig 1): Balgzand (N 52.92824, E 4.82349), Texel (N 53.12321, E 4.90133), Schiermonnikoog (N 53.46979, E 6.17340) and Uithuizen (N 53.46632, E 6.68830). Site selection was based on seagrass habitat suitability models [21, 33] and seed dispersal models [29, 34, 35]. Apart from their apparent potential high suitability for seagrass growth, restoration sites were all exposed at low tide for 5–6 hours and varied in sediment type from fine sand to muddy. All restoration sites lacked *Z*. *marina* presence prior to the first seed-based restoration trials in 2011 [36]. In the summer of 2014, very small densities of *Z*. *marina* (all <1% cover) were observed across a limited extend of the restoration sites prior to seeding: 0.04 ha on Balgzand, 1.32 ha on Uithuizen, 0.72 ha on Schiermonnikoog, 0.0 ha on Texel [37]. All seeding experiments were conducted outside areas where plants were encountered. Because some sites had no or extremely low plant numbers in the summer of 2015 (Table 1, Balgzand and Texel, for detailed description see below), restoration experiments in successive years only focused on the most successful sites (Uithuizen, Schiermonnikoog).

**Table 1:** Overview of restoration activities in four subsequent years (2014–2017) on five sites in the Dutch Wadden Sea.

Location	Year	Period of Seeding	Size seeded area (m^2^)	Seeding method	Biomass seeded (kg)	# Seeds seeded	Monitoring period	Monitoring Method	# Total number of plant	Surface area with plants (ha)	Plant density (# m^-2^)	% seed loss
Balgzand	2014	Fall	5760	BuDS	22.5	190,000	-	-	-	-	-	-
	2015	-	-	-	-	-	Summer	BCPD	14	0.1	0.014	99.992
	2016	-	-	-	-	-	-	-	-	-	-	-
	2017	-	-	-	-	-	-	-	-	-	-	-
Schiermonnikoog	2014	Fall	5760	BuDS	22.5	190,000	-	-	-	-	-	-
	2015	Fall	200	BuDS in frame	132	780,000	Summer	BCPD	995	15	0.007	99.476
	2016	-	-	-	-	-	Summer	BCPD	2076	5.6	0.037	99.734
	2017	-	-	-	-	-	Summer	BCPD	759	13	0.006	-
Uithuizen	2014	Fall	5760	BuDS	22.5	190,000	-	-	-	-	-	-
	2015	Fall	200	BuDS in frame	132	780,000	Summer	BCPD	406	3.5	0.012	99.937
	2016	-	-	-	-	-	Summer	BCPD	831	5	0.017	99.893
	2017*	Spring	2640	DIS	NA	600,000	Summer	Ground counts	1546*	0.26*	0.590*	99.742
Texel	2014	Fall	20,320	BuDS	318	2,686,000	-	-	-	-	-	-
	2015	-	-	-	-	-	Summer	BCPD	0	0	-	100.00
	2016	-	-	-	-	-	-	-	-	-	-	-
	2017	-	-	-	-	-	-	-	-	-	-	-

Seeding was performed in either Fall or Spring. Seeding methods are abbreviated: BuDS stands for Buoy Deployed Seeding and DIS stands for Dispenser Injection Seeding. Number of seeds seeded are truncated to the nearest 10000. Note that except for UH17, seeding was generally conducted in fall, whereas monitoring was conducted in the preceding year in summer. The result from the 2017 seeding experiment on Uithuizen (indicated by *) are also separately displayed in Fig 4. Uithuizen 2017 numbers displayed in this table are only counted within plots. Since many plants also settled outside the plots, but were not counted, total seed losses could not be determined. BCPD is the abbreviation for ‘Batcheler Point Corrected Distance’ monitoring method.

#### Donor site and seed collection

**Fig 2:**
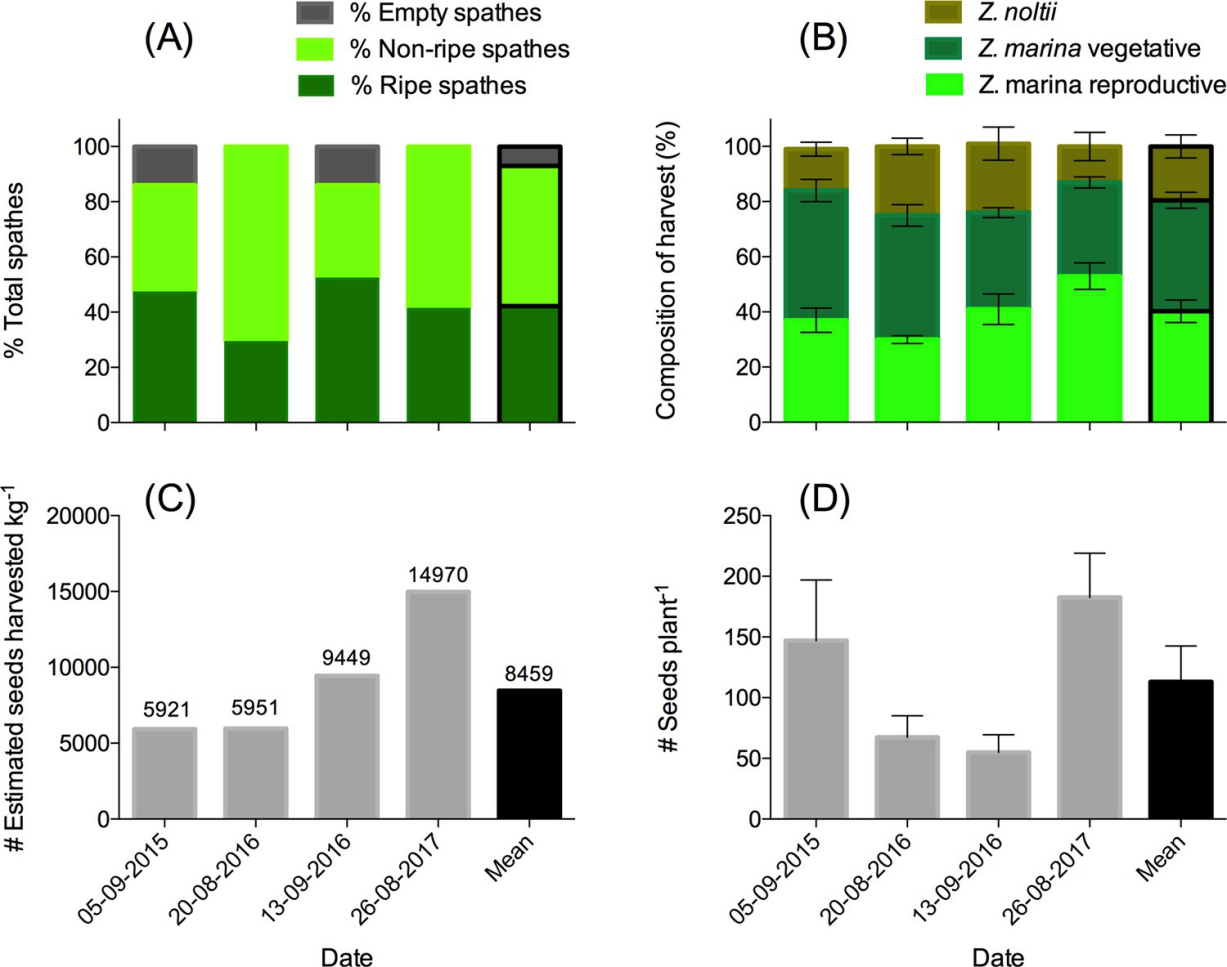
Measurements from the seed donor site on Sylt. (A) Spathe composition per plant with spathes containing ripe seeds at time of harvest (% ripe spathes), spathes containing flowers or developing seeds (% non-ripe spathes) and spathes which have already shed seeds (% empty spathes), (B) Composition of harvested seagrass material divided into seed-bearing spathes of Z. marina, vegetative (leaf, rhizome, root) material of *Z*. *marina*, and accidentally harvested *Z*. *noltii* (whole plant) (n = 4). (C) Number of seeds per harvested kilogram estimated based on measurements on composition of harvest material, the weight per spathe, ration spathes containing ripe/non-ripe seeds and number of ripe seeds/spathe and (D) number of seeds plant^-1^ during harvest (n = 10).

Seed material (*Z*. *marina*) for restoration experiments was collected on Sylt (Puan Klent, N 54.7831, E 8.29487) in the Northern German Wadden Sea (Fig 1). Seed-bearing shoots (spathes) were collected by a group of citizen volunteers (NGO Natuurmonumenten), from a healthy mixed *Z*. *marina*/*Z*. *noltii* meadow [19] with a mean *Z*. *marina* plant density of 26 ± 10 plants m^-2^. The material was collected yearly (2014–2017) between 20 August and 15 September. These dates were determined based on the amount of spathes containing ripe seeds (>40%, Fig 2A). Although seed-bearing shoots were targeted for harvest, these comprised only 40 ± 4% of the harvested material (Fig 2B), while the other 60% consisted of vegetative parts of *Z*. *marina* and *Z*. *noltii* (Fig 2B). This composition did not differ significantly between moments of harvesting. Yearly, a total amount of 200–400 kg of seagrass material (~15,000–30,000 *Z*. *marina* plants) was harvested from the donor site, equaling <1% of the plants growing at the >50 ha donor site. Each kilogram of material contained an average of 75 plants that each contained 113 ± 30 seeds (Fig 2D), yielding an estimated 8459 ± 2134 (n = 4) seeds on average per kilogram (Fig 2C). Harvested seagrass material was transported to the Netherlands under refrigerated (~7°C) conditions, within a day after harvest.

### Seeding methods

#### 2014. buoy deployed seeding

**Fig 3:**
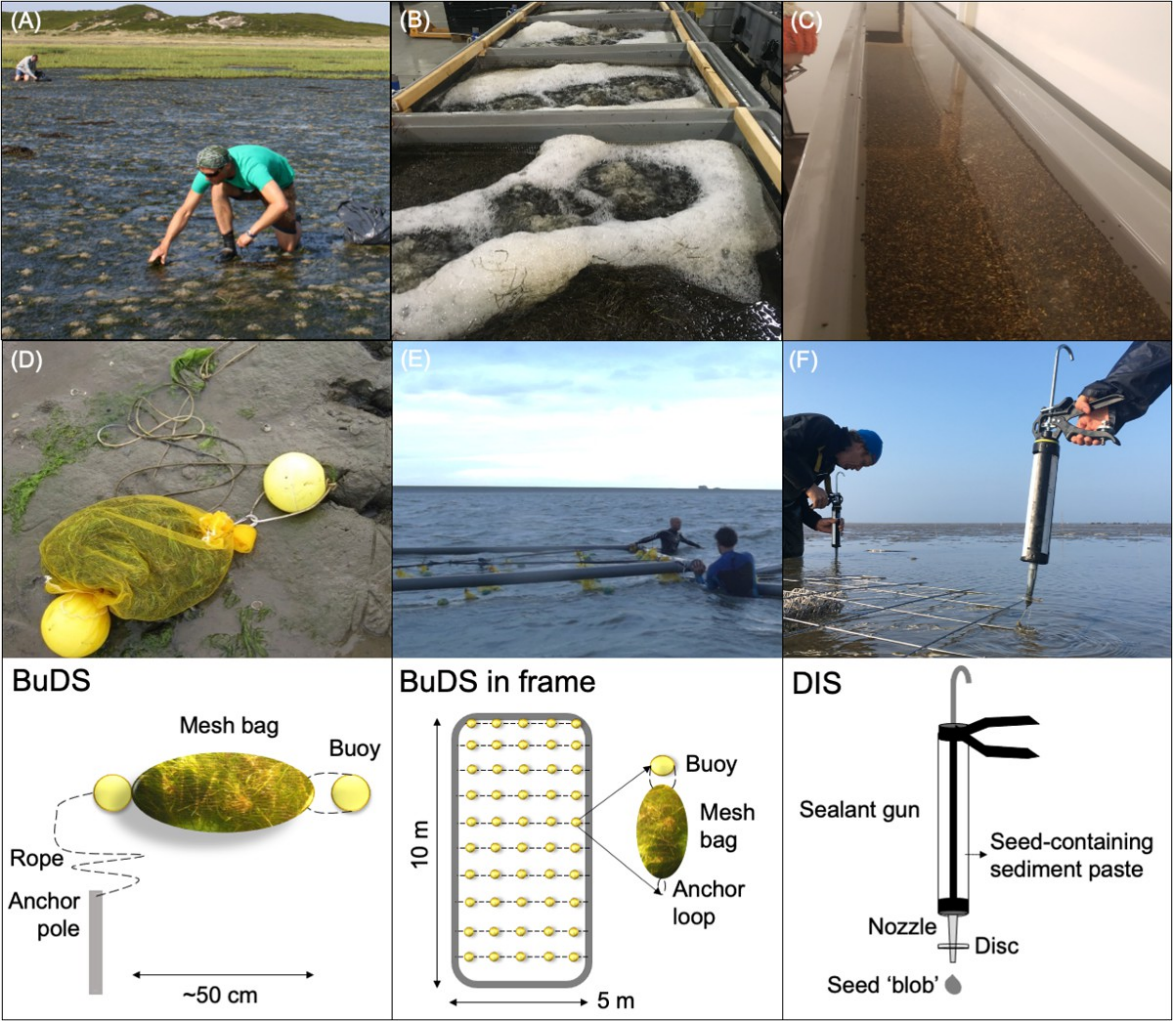
Visual overview of methods. (A) volunteer is selectively harvesting seed-bearing eelgrass shoots from the donor site on Sylt, (B) Harvested seed-bearing shoots are kept in aerated seawater tubs until all the seeds have dropped out of the spathes, (C) seeds are stored under controlled conditions with flow-through system, (D) Buoy Deployed Seeding (BuDS) in the intertidal, harvested material is put in a mesh bag (photo, illustration), (E) Buoy Deployed Seeding in frame (BuDS-in-frame) in the field (photo) and birdeye view (illustration), (F) Dispenser Injection Seeding (DIS) on the intertidal mudflats in spring (photo, illustration).

Since scale-dependent self-facilitating processes appear to be important for seagrass restoration [8, 38], we first attempted large-scale seagrass restoration using the BuDS-method [28], which was adopted from previous seed-based restoration attempts in 2011 and 2012 [27]. Seed-based seagrass restoration is gaining traction as a restoration method [39–41], as it is cost-effective [42], can be easily upscaled [28], and ensures maintenance of desirable high genetic diversity [43–45]. For the BuDS-method, 800 g of harvested seed-bearing shoot material (unprocessed, also containing vegetative *Z*. *marina* and *Z*. *noltii*) was placed in 2 mm mesh bags (nylon ‘potato bags’) with a small buoy. Filled mesh bags were subsequently tied to another buoy (Fig 3A), connected to a 4 m long rope that was securely anchored in the mudflat by a 0.7 m long PVC pole. The poles were placed in such a way that an estimated surface area of 5760 m^2^ was seeded by the movement of the mesh bags on the ropes. On Texel, this surface area was increased to 20,320 m^2^ by deployment of more mesh bags. An estimated 190,000 seeds were seeded per site, except for Texel where 2,686,000 seeds were seeded (Fig 1, Table 1). BuDS-units were deployed within 5 days after seed harvest, and were left out in the field for 8–10 weeks.

#### 2015. Buoy deployed seeding in frame

To enhance seeding densities, we decided to adapt the method of seeding in 2015 and test it at the most successful sites from 2014 –Uithuizen and Schiermonnikoog–(Fig 1). Rather than being tied to poles, BuDS were tied to horizontal ropes spaced 1 m apart within four PVC frames of 5x10 m (Fig 3E), covering a total surface area of 200 m^2^. The frames were deployed at the site during high tide, where they were submerged by filling them with water, and securely anchored to the sediment. This method reduced the seeding circle of the bags to around 1 m^2^. Each frame contained 11 rows with six BuDS (66 per frame, 264 per site) that were filled with 500 g harvested seagrass material. Thus, a total of 132 kg harvested seagrass material, containing an estimated 780,000 seeds (Table 1), was seeded per site.

#### 2016–2017. Processing harvest and controlled seed storage and ‘Dispenser Injection Seeding’ (DIS)

As both BuDS methods resulted in high seed loss in winter, we moved on to alternative methods aimed at storing seeds inside during winter to reduce both winter losses through hydrodynamics and losses from *Phytophthora* spp. infection [46]. Hence, for the 2017 seeding experiments, all harvested seagrass (420 kg in total) was processed after harvest. Harvested material was deposited in big, 1 mm mesh sieves hanging in strongly aerated seawater tanks (max 21 kg/ 500 L) with flow-through system (Fig 3B). Seawater originated from the nearest Wadden Sea harbour (Lauwersoog, N 53.41177, E 6.19918) and had an average salinity of around 27 ppt (Rijkswaterstaat Waterdata). Harvested material was left in the seawater for a maximum of 5 weeks and all debris that had sunken through the sieves to the bottom (including seeds) was collected on a weekly basis and then processed to separate seeds from mud snails (*Peringia ulvae*) and dead organic matter [47]. Cleaned-up seeds were subsequently stored in 4 m x 10 cm roof gutters filled with a 2-cm layer of aerated, and a constant flow-through of artificial seawater (30 ppt, Tropic Marin ©) (Fig 3C). Seeds were spread out across the gutters (five in total) to minimize the seed layer depth (max 0.5cm) to prevent anoxia [36]. This set-up was located in a cool (4°C) and dark climate-controlled room. Seeds were additionally treated with a 0.2 ppm copper sulfate treatment to reduce infection by *Phytophthora* spp. and *Halophytophthora* spp. [48]. The total harvested seagrass material (420 kg) contained an estimated 2.5 million seeds. However, we stored only about 1.2 million seeds, indicating a loss of about 47% due to seed-processing. Stored seeds were also checked for viability using germination tests [46] and 92 ± 4% of the stored seeds were tested as viable. Viability was not checked for seeds that were directly transferred to the field in 2014 and 2015. Additional test-planting of seeds in a mesocosm with mudflat sediment was conducted with or without fresh water (1h, 0 ppt DI) water pre-treatment to stimulate germination [49], to test additional expected viability in the field. This yielded seed germination rates (as counted by the emergence of green seedlings) of 42 ± 27% (no freshwater treatment) up to 79 ± 25% (freshwater treatment), indicating high seed quality.

After winter seed storage, we needed to develop a new method to disperse seeds into the field in spring. We therefore developed the DIS-method. For this method, seeds were injected into the sediment in a blend of seeds and thickened mudflat sediment. Mudflat sediment (median grain size of 24 μm, 15% OM), originating from a nearby natural seagrass meadow, was collected for this purpose. Next, this sediment was sieved over a 1 mm mesh to remove benthic animals and large particles. Subsequently, the sieved sediment was left in a fine sieve (1mm mesh) overnight to reduce the water content by using gravity to release some moisture from the sediment through the mesh. This resulted in a thickened, aerobic sediment paste that was stored at 6°C until seeding. Before seeding, a set volume of sediment paste was homogeneously mixed with a fixed number of seeds (measured by volume), to be able to obtain target seed densities when injecting. Seeds were soaked in freshwater (0 ppt) for 24 h before being added to the mixture [50]. This mixture was used to fill-up 310 mL sealant tubes, which were loaded into sealant guns (Fig 3F). Sealant guns were tuned and seed ‘blobs’ were subsequently injected at a set depth of 2 cm, by adding a plastic disc to the nozzle to mark the required depth (Fig 3F, ***S1 Movie***).

#### 2017. Plot size and seed density experiment

In 2017, we set-up a small-scale, full-factorial experiment to test the DIS-method and the effects of plot size (two levels: 200 and 20 m^2^) crossed with number of seeds injection^-1^ (two levels: 2 and 20 seeds injection^-1,^ resulting in a seed density of 40 or 400 injected seeds m^-2^, the error margin was estimated to be 10%). This resulted in four treatments with six replicates each. Seeds were injected early April, with a density of 20 injections m^-2^. Thus, seed density treatments received the same number of injections, but had more seeds injection^-1^. Plots were 14.10 x 14.10 m (200 m^2^) or 4.5 x 4.5m (20 m^2^). The experiment was set-up on the restoration site that was consistently the most successful based on previous years’ results (Uithuizen, Fig 1). An estimated number of 600,000 seeds were applied in this experiment. The rest of the estimated 1.2 million seeds were applied in other experiments (mesocosm and field experiments).

#### Monitoring: Batcheler-Corrected Point Distance method

Monitoring was conducted once per growing season in August. Intertidal seagrass monitoring in the Dutch Wadden Sea, commissioned by the government, is performed every 3 years, and uses percent cover to report seagrass presence and density. Since almost all seagrass densities are below 1% cover [19], we performed additional monitoring of our restoration sites based on the Batcheler-Corrected Point Distance (BCPD) method [51, 52] to estimate the plant density and the overall population size. For this method, the distance from starting points along a transect to their nearest object is measured, followed by a measurement from that object to the next nearest object, and again, from that second object to its next nearest object (S1 Fig, [53]). We first mapped the spatial extent of the seagrass-inhabited area, using a handheld GPS; the outer border was drawn where the distance between plants fell below 30 m. Next, we set-up two perpendicular transects across the mapped area, with a Batcheler-Corrected Point Distance starting point every 20 m along the transect. From each transect point, we searched for plants within a 10 (in 2016–2017) to 15 (2015) m radius circle, with the diameter depending on the plant density [50]. After the first plant (r_p_) was found we repeated this procedure for the second (r_n_) and third (r_m_) plant if possible. Plant densities (# m^-2^) were calculated as total plant number / total area with plants (m^2^) [53]. Plant densities rather than shoot densities were monitored due to the annual morphology of intertidal eelgrass in the Wadden Sea: plants grow in clumps, investing in seed production rather than rhizome elongation. In addition, plant counts provided more information on population demographics compared to shoot counts. In 2017, plant densities were higher and spread out across a smaller surface area, rendering the BCPD method useless. We therefore counted all plants per plot instead. Since our target species, annual *Z*. *marina*, cannot build up a seed bank >1 year, plant counts were determined to be representative of results from seeding trials in the same year.

#### Comparison of restoration methods

To compare restoration methods (i.e. BuDS, BuDS in frame and DIS), we compared plant densities and seed losses between 2015–2017 from our Uithuizen site, which was the only site which was repetitively seeded throughout this period. Plant densities were determined using the two different monitoring methods indicated above: the Batcheler-Corrected Point-Distance Method for 2015 and 2016 and plant counts per plot for 2017. Plant densities in 2015/2016 were inferred from the Batcheler-Corrected Point-Distance Method that yielded total plant numbers per site by #total plant count / total areal extent with plants (m^2^). In 2017, plant densities were determined per plot by #total plant count per plot / plot size (m^2^). Percentage seed loss as reported in Table 1 were also determined in two different ways. In 2015 and 2016, seed losses were calculated as 100-(#total adult plants monitored on a restoration site / # total seeds applied to that site) *100%. In 2017, Percentage seed loss was calculated per plot as 1- (#total adult plants counted plot^-1^ / #injected seeds plot^-1^) * 100%. In addition, we report establishment rates, which were inferred from seed losses and calculated as 100 –(% seed loss).

#### Statistical analyses

We made a comparison between the two BuDS methods by comparing the plant density and seed loss results from 2015 and 2016 using chi-square tests. Differences in composition of harvest between moments of harvest and seed numbers per plant were tested using separate (*Z*. *marina* spathes, *Z*. *marina* vegetative and *Z*. *noltii*, seeds plant^-1^) linear mixed models (lme, nlme package) with “moment of harvest” as a fixed factor and “year” as a random factor. Results (plant densities, seed loss) from the 2017 experiment were also tested using linear mixed models with “density” and “plot size” as fixed factors (interactively tested) and “block” as a random factor. Model assumptions were checked on model residuals using a Shapiro-test and by visually inspecting qq-plots and histograms. All statistical tests were performed in R version 3.5.1 (© 2021 the R Foundation for statistical computing).

### Ethics statement

Permits for *Z*. *marina* seed collection on Sylt were obtained (verbal approval) from the Alfred Wegener Institute (AWI) on Sylt. Permission to conduct seagrass (*Z*. *marina)* restoration experiments was obtained (written approval) from the Province of Fryslân, the Netherlands. In addition, local nature managers (Natuurmonumenten, Landschap Noord-Holland) provided verbal consent for conducting these experiments on their land.

## Results

### 2015. BuDS

Although three of our experimental sites were seeded with the same amount of seeds in 2014 (190,000 seeds per 5760 m^2^ equaling 33 seeds m^-2^), we found great differences in plant numbers among sites in 2015. Total plant numbers per site were generally low, ranging from 14 (Balgzand) to 406 (Uithuizen) and 995 (Schiermonnikoog). On Texel, where 132 seeds m^-2^ were seeded, plants were completely absent (Table 1).

On sites with plants, plants were distributed across a large area (3.5–15 ha), resulting in extremely low densities (0.007–0.012 plants m^-2^). These results indicate high seed loss (99.84–100%), very likely due to seeds being washed away by waves and currents, apparent from the large area where adult plants were found.

### 2016. “BuDS-in-frame”

In 2015, the “BuDS-in-frame” method was applied, seeding 780,000 seeds per 200 m^2^ (~3900 seeds m^-2^). This method yielded on average 2x higher plant densities in 2016 (Χ^2^, *P <* 0.001) than the regular BuDS method in 2015: 831 plants in Uithuizen and 2076 plants on Schiermonnikoog (Table 1). The actual seeded area in 2016 (5.6 ha Schiermonnikoog, 5.0 ha Uithuizen) was significantly lower compare to 2015 (Χ^2^, *P =* 0.011). In addition, plant densities did not increase significantly (Χ^2^, *P =* 0.921), and were still far below target densities (>10 plants m^-2^) with only 0.017 and 0.037 plants m^-2^ on Uithuizen and Schiermonnikoog respectively (Table 1). Seed loss was also still very high: 99.73–99.89%. These results indicate that the adapted “BuDS-in-frame” method was inadequate to reach our restoration targets.

### 2017. Scale and seed density experiment

**Fig 4:**
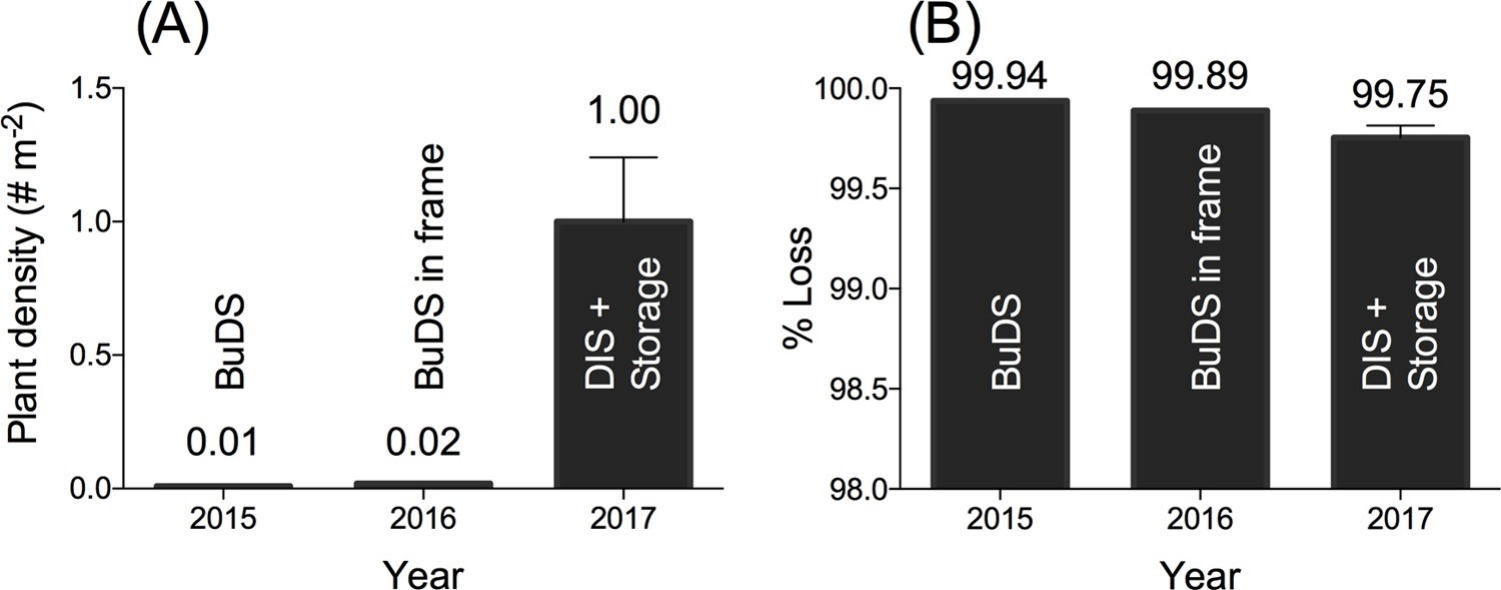
Comparison results Uithuizen experiment (2017) with results of previous (2015–2016) years. (A) Plant densities (m^-2^) (B) Loss rates (%).

**Fig 5:**
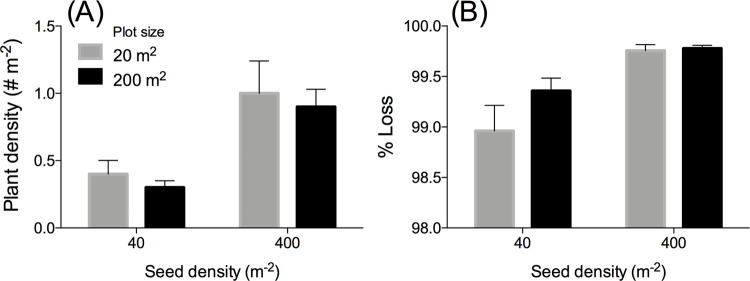
Results restoration experiment Uithuizen 2017. (A) Mean plant densities (m^-2^) and (B) Loss rates (%) from seeds to seed-bearing plants.

In 2017, we switched from the BuDS methods to the controlled seed storage over winter and DIS-seeding in spring. This method yielded up to 100x higher plant densities than in previous years (Up to a max. of 1.8 plants m^-2^, Fig 4). Plot size (20 vs. 200 m^2^) did not affect plant densities (Fig 5), but high seed density plots yielded 3x higher plant densities (1.0 plants m^-2^) than low seed density plots (0.33 plants m^-2^, Linear Mixed Model, F_1,15_ = 21.357, *P* < 0.001). Since high seed density plots received 10x more seeds (40 vs. 400 seeds m^-2^), plant densities in the high seed density plots were actually >3x lower than expected. Although absolute plant numbers were higher in the high seed density plots, the low-density plots experienced significantly (3.6x, Linear Mixed Model, F_1,15_ = 17.4, *P* < 0.001) lower seed loss than the high-density plots (99.2 vs. 99.8% respectively), indicating that every 1000 seeds seeded yielded 8.4 and 2.3 plants in the low- and high-density plots respectively. Plot size did not affect seed loss. Although seed loss in this experiment was reduced in this experiment compared to previous years at this location (2015 & 2016, Table 1), average seed loss was still >99% (except for three individual plots).

## Discussion

Attempts at seagrass restoration around the world have been challenging and successes have been linked to large-scale planting attempts [8, 9, 54]. We deployed multiple seed-based restoration experiments targeting intertidal annual *Z*. *marina* in the Dutch Wadden Sea in 2014–2017. Despite early set-backs, an adaptive approach using yearly advancing insights allowed us to develop a new seed-based restoration technique, the DIS-method. Due to the storage of seeds during winter and seed out-planting using this method, we managed to produce 100x higher plant densities within two years (0.012 to 1.8 plants m^-2^). This new method needs further improvement, but shows great promise for successfully upscaling seed-based seagrass restoration. Previously used methods (2014/2015), BuDS and ‘BuDS in frame’, were deemed unsuitable at our restoration sites as these methods resulted in very low (0.007–0.037 plants m^-2^) plant densities and high seed losses (>99.48%). Our results indicate that despite early setbacks in restoration success, an adaptive, research-based approach focused on technological advancement allows for taking successful steps towards understanding key-processes for seed-based seagrass restoration in intertidal areas.

### Comparison of restoration methods

Interest in seed-based seagrass restoration is growing, as it has some major benefits in comparison with other planting techniques. It is cost-effective, allows for maintaining high genetic diversity and enables upscaling of restoration attempts [39–42, 44, 55].

A variety of seagrass seeding methods have been developed, including hand-broadcasting [42], a mechanical seed planter [56], the use of protective bags [57], and buoy-deployed seeding techniques [28].Most of these methods found relatively low seedling establishment rates in the field, with a maximum of 20% [58], but commonly as low as 1% [57, 59]. Although we could not distinguish between seedling settlement and plant settlement, we found even lower plant establishment rates (<0.5%) on all our intertidal study sites. We assume that most losses that occurred in our study area occurred in the seed-phase as several studies have shown that if seeds are retained in suitable spots, seedling establishment and development is generally high [14, 60, 61]. Our two BuDS-methods (regular and “BuDS-in-frame”) yielded plant establishment rates lower than 0.1%. We considered this to be problematic for restoration success since we were targeting annual plants that completely depend on successful seedling establishment every single year. Furthermore, to maintain a self-sustaining population, a >0.9% plant establishment would be needed based on measurements on seed production of donor plants on Sylt (mean 113 seeds plant^-1^). Next to low plant establishment rates, we also found extremely low total plant numbers in our BuDS-trials (Table 1). Surprisingly, the BuDS-method did result in seed dispersal across a large areal extent, as opposed to findings from previous studies that mostly reported short-range seed transport after BuDS-deployment [28, 39].

In contrast to the BuDS-method, that led to very high seed losses and low establishment success rates, the DIS-methods yielded far more promising results in our study system (intertidal Wadden Sea). The DIS-method yielded promising results in terms of plant densities (mean of 1.0 and max. 1.8 plants m^-2^ in the highest seed density treatment), as compared to 2015 and 2016 (BuDS-methods, 0.01 and 0.02 plants m^-2^ respectively). However, plant-establishment rates with this method were still low (0.23–0.84%), but maximum seed establishment rates were as high as 1.62%. The DIS-method is thus a promising method to reach target plant densities for intertidal *Z*. *marina* restoration, but the method should still be improved in terms of optimal planting depth, # seeds inject^-1^ and # injects m^-2^ to optimize both plant densities and establishment. After optimization, this method (hand- or machine-labour) might even enable practitioners to upscale restoration attempts of *Z*. *marina*, which is vital to restore positive density-dependent feedbacks [8]. With further research on mechanisms of seed losses, this method may also be adapted for seed-based restoration of other seagrass species.

Key to the success of the DIS-method is the controlled storage of seeds during the winter prior to spring seeding. In the field, high winter seed losses may have resulted from bioturbation [62–64], seed predation [65], disease [46], high sediment mobility [66] and waves and currents moving seeds into unsuitable growth conditions [67]. Winter storage has resulted in high seed viability rates (>75%), successfully keeping seeds away from all bottlenecks listed above except for disease (i.e. *Phytophthora* spp. infection), which we reduced by treating stored seeds with copper [48].

### Scale and density

van Katwijk, Thorhaug [8] found that large-scale plantings generally yielded higher restoration success. This may be due to the ecosystem engineer-properties of seagrasses: seagrasses are able to manipulate their own biotic and abiotic environment, which enables them to improve their own growing conditions with increasing densities [38, 68]. For instance, by alleviating hydrodynamic stress, by attenuating waves and currents and by stabilizing the sediment with their dense root mats [69, 70]. Secondly, the scale of restoration may be important to deal with environmental stochasticity of dynamic coastal habitats [71]. In our 2017 experiment, we did not observe a positive scale effect on plant densities (Fig 5): large-scale plots (200 m^2^) did not yield higher plant densities than small-scale plots (20 m^2^). This lack of positive scale effects may be due to the overall low plant densities that do not enable self-facilitating density-dependent feedbacks to operate. Despite our low densities, our findings are in line with the findings of Orth, Fishman [72] who found no scale-effect in seed germination and seedling establishment in Chesapeake Bay. However, we did find negative effects of high seed densities on plant establishment as we only found 3x higher plant establishment while we had planted 10x more seeds (40 vs. 400 seeds m^-2^). This may be due to our seeding method (DIS), where we obtained high seed densities by increasing the number of seeds per injected ‘blob’ (2 or 20 seeds blob^-1^) rather than increasing seed density by increasing the number of injections m^-2^. This may actually have facilitated intraspecific competition rather than facilitation as was found by Granger, Traber [73]

Next to the lack of density-dependent facilitation, low plant densities may also strongly affect population dynamics of a restored annual seagrass population. These populations completely depend on successful fertilization, seed production and seed retention for the establishment of next year’s generation. Low plant densities may prevent successful fertilization of flowers [74], although pollination may occur up to 70 m away [75]. Additionally, self-pollination does occur in *Z*. *marina* [76], but may decrease genetic diversity through inbreeding at the risk of extinction [43]. Low plant densities may also negatively affect seed retention through reduced structural complexity of intertidal soft-bottom systems that usually aids *Z*. *marina* seed retention [77]. Thus, positive effects of scale-dependent intraspecific facilitation may be context-dependent and alternative facilitation strategies, e.g. interspecific facilitation, could also be explored.

### Environmental suitability

The first step in ecological restoration is to reduce existing pressures (either anthropogenic or natural) on a species, population or ecosystem, before engaging in active restoration. Thus, causes of degradation need to be assessed and removed to ensure restoration success [78, 79]. Despite our relative increase in the restoration successes of intertidal eelgrass (yielding 100x higher plant densities between 2015–2017), absolute plant numbers were still very low (max. 1 plant m^-2^) and seed losses extremely high (>99.75%). This may not only be due to limitations in restoration techniques, but could also result from insufficient environmental suitability of the selected restoration sites. Natural intertidal seagrass beds have all but disappeared from the Dutch Wadden Sea [19], very likely due to an accumulation of stressors such as eutrophication, increased sediment dynamics and bioturbation [14–17]. Despite a strong improvement in water quality since the 1980s [80], natural recovery of intertidal seagrass (*Z*. *marina* and *Z*. *noltii*) has not occurred. This may be an indication of an ongoing low environmental suitability, or a general lack of donor material to support recovery. However, despite actively providing donor material through restoration experiments, most of our selected sites turned out to be unsuitable for the restoration of intertidal *Z*. *marina*. This is surprising, because site selection was based on multiple habitat suitability models that took the most important determinants for intertidal seagrass presence into account (e.g. nutrients, sediment characteristics, bathymetry, hydrodynamics, salinity) [21, 33]. This indicates that some unaccounted factors may be present, e.g. seed predation, pathogens, bioturbation or others, yielding sites unsuitable. Thus, next to advancing restoration methods, a careful evaluation of environmental habitat suitability is needed to warrant restoration success [81].

### Implications and recommendations

Seagrass restoration is challenging and success rates are generally low [8]. Although large-scale scale planting generally improves restoration success [8], large-scale efforts using the BuDS-method did not lead to restoration success (in density and plant numbers) in our trials in the Dutch Wadden Sea. We conclude that the BuDS method, and the follow-up “BuDS-in-frame” method are unsuitable for the intertidal Wadden Sea due to high seed loss rates due to waves and currents, bioturbation and disease. Our newly developed DIS-method, combined with controlled seed storage, seems like a promising technique to improve restored plant densities in our intertidal study area. This method however still needs some fine-tuning to optimize results and to allow for large-scale planting. Additionally causes of seed loss should be further investigated to further enhance restoration successes. Finally, even though we experienced major setbacks in our seed-based eelgrass restoration project in the intertidal Wadden Sea, we recommend an adaptive, research-based approach focused on technological advancement, to improve restoration. This may aid in taking small, but successful steps towards seagrass restoration in intertidal areas.

## Supporting information

S1 Fig Illustration of the Batcheler-Corrected Point Distance monitoring method. (DOCX)

S1 Movie Dispenser Injection Seeding (DIS) in action. (MOV)
