# Supplementary material for: Adaptive intertidal seed-based seagrass restoration in the Dutch Wadden Sea
Source: PLoS One. 2022 Feb 9;17(2):e0262845. doi: 10.1371/journal.pone.0262845 (PMC8827467; doi:10.1371/journal.pone.0262845)
Supplement: S1 Fig — (DOCX) [file pone.0262845.s001.docx]

**Supporting information**


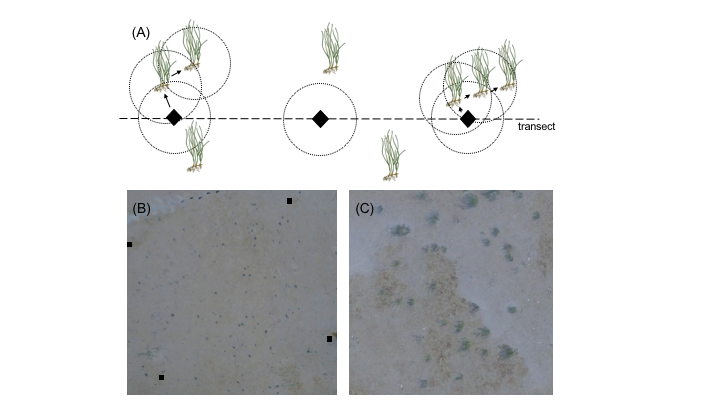


**Figure S1.** Visual overview of the Batcheler-Corrected Point Distance (BCPD) method, circles indicate searching radius adapted from Rempel et al. 2012.
